# *Elizabethkingia bruuniana* Infections in Humans, Taiwan, 2005–2017

**DOI:** 10.3201/eid2507.180768

**Published:** 2019-07

**Authors:** Jiun-Nong Lin, Chung-Hsu Lai, Chih-Hui Yang, Yi-Han Huang, Hsi-Hsun Lin

**Affiliations:** E-Da Hospital, Kaohsiung, Taiwan (J.-N. Lin, C.-H. Lai, Y.-H. Huang);; I-Shou University, Kaohsiung (J.-N. Lin, C.-H. Lai, Y.-H. Huang);; Meiho University, Pingtung, Taiwan (C.-H. Yang);; Taipei Veterans General Hospital, Taipei, Taiwan (H.-H. Lin)

**Keywords:** *Elizabethkingia bruuniana*, *Elizabethkingia*, clinical characteristics, 16S rRNA, *rpoB*, bacteria, Taiwan, phylogeny, DNA–DNA hybridization, average nucleotide identity, fluoroquinolone resistance, quinolone resistance–determining regions, drug susceptibility, antimicrobial resistance, antimicrobial susceptibility

## Abstract

Using 16S rRNA and *rpoB* gene sequencing, we identified 6 patients infected with *Elizabethkingia bruuniana* treated at E-Da Hospital (Kaohsiung, Taiwan) during 2005–2017. We describe patient characteristics and the molecular characteristics of the *E. bruuniana* isolates, including their MICs. Larger-scale studies are needed for more robust characterization of this pathogen.

The *Elizabethkingia* genus comprises gram-negative, aerobic, nonmotile, nonspore-forming, nonfermenting rod-shaped bacteria (*1*). This genus previously comprised *E. meningoseptica*, *E. miricola*, and *E. anophelis*. In August 2017, Nicholson et al. proposed adding 3 new species, namely *E. bruuniana*, *E. ursingii*, and *E. occulta*, to this genus (*1*). However, little information exists about these species. In this study, we report the clinical characteristics and demographics of a group of patients with *E. bruuniana* infection in Taiwan and the molecular features of their *E. bruuniana* isolates.

We conducted this study at E-Da Hospital, a 1,000-bed university-affiliated medical center in Kaohsiung, Taiwan; this study was approved by the institutional review board of the hospital (no. EMRP-106-105). We searched the hospital database to identify microbial cultures performed during January 2005–December 2017 that yielded *Elizabethkingia*. The isolates were initially identified by staff in the clinical microbiology laboratory using API/ID32 phenotyping kits or VITEK MS (both from bioMérieux, https://www.biomerieux.com). We reidentified these species as *Elizabethkingia* using both 16S rRNA and *rpoB* gene sequencing. The primers and methods we used for amplification and sequencing of the 16S rRNA and *rpoB* genes were described previously (*1*,*2*). We compared the assembled 16S rRNA gene sequences with the nucleotide sequences of *Elizabethkingia*-type strains present in GenBank. We considered isolates with >99.5% similarity in the 16S rRNA gene sequence members of the same species, as recommended in a previous study (*3*). We constructed a phylogenetic tree using the *rpoB* genes of the isolates exhibiting >99.5% 16S rRNA gene sequence identity with the *E. bruuniana* type strain G0146^T^. We calculated the average nucleotide identity using OrthoANI (*4*) and computed in silico DNA–DNA hybridization (DDH) using the Genome-to-Genome Distance Calculator (*5*), using the average nucleotide identity value of >95% and the DDH value of >70% separately as criteria for species delineation (*4*,*5*). We sequenced the quinolone resistance–determining regions of DNA gyrase (*gyrA* and *gyrB*) and topoisomerase IV (*parC* and *parE*) to look for mutations associated with resistance (Appendix Table).

For the 13-year period, we found 103 nonduplicate *Elizabethkingia* isolates in the database of the clinical microbiology laboratory. Among these, 8 isolates shared >99.5% 16S rRNA gene sequence identity with *E. bruuniana* G0146^T^, and an *rpoB* gene–based phylogenetic analysis revealed that 6 of the 8 isolates were more closely related to *E. bruuniana* G0146^T^ (Appendix Figure 1). We previously published the complete whole-genome sequence of 1 of these 6 isolates, EM798-26 (GenBank accession no. CP023746) (*6*). Using 16S rRNA gene sequence analysis, we initially identified this isolate as *E. miricola*. Average nucleotide identity analysis demonstrated that EM798-26 and *E. bruuniana* G0146^T^ share 97.7% whole-genome similarity (Appendix Figure 2). Using in silico DDH analysis, we predicted a DDH value of 81.7% for EM798-26 and *E. bruuniana* G0146^T^ (Appendix Figure 3). These results support that EM798-26 and the other 5 isolates (EM20-50, EM455-89, EM828-05, EM863-68, and EM891-63) are *E. bruuniana*.

These 6 isolates were collected from 6 (4 male and 2 female) patients (Table) with a mean age of 71.7 (SD +11) years. The sources of isolation included bronchoalveolar lavage fluid (n = 2), blood (n = 2), urine (n = 1), and the tip of the central venous catheter (n = 1). All infections were healthcare associated. Two patients had septic shock, and all patients had >1 concurrent medical condition, such as hypertension, diabetes mellitus, or a malignancy. Antimicrobial therapy included piperacillin/tazobactam, trimethoprim/sulfamethoxazole, levofloxacin, or tigecycline, either singly or in combination. None of the patients died of *E. bruuniana* infection.

**Table:** Characteristics of patients infected with *Elizabethkingia bruuniana*, Taiwan, 2005–2017, and antimicrobial susceptibility of the *E. bruuniana* isolates*

Category	Patient no./isolate no.					
	No. 1/EM20-50	No. 2/EM455-89	No. 3/EM798-26	No. 4/EM828-05	No. 5/EM863-68	No. 6/EM891-63
Patient characteristics						
Year of illness	2005	2011	2015	2016	2016	2017
Age, y/sex	67/F	62/F	81/M	60/M	88/M	72/M
Site of microbe isolation	Urine	CVC tip	Blood	Blood	BAL fluid	BAL fluid
Clinical manifestations	Urinary tract infection	Septic shock	Primary bacteremia	Primary bacteremia	Pneumonia	Pneumonia, septic shock
Underlying conditions	Cervical cancer, hypertension	Maxillary osteosarcoma, hypothyroidism, hypertension	Lymphoma, chronic kidney disease	Brain meningioma, stroke, hypertension	Liver cirrhosis, hypertension, CHF	Esophageal cancer, diabetes mellitus, hypertension
Treatment	TMP/SMX	TZP	Levofloxacin	TZP, levofloxacin	Levofloxacin	Tigecycline, levofloxacin
Outcome	Survived	Survived	Survived	Survived	Survived	Survived

MIC, mg/L†						
Piperacillin	64	64	>64	64	>64	64
TZP	32/4	>128/4	32/4	32/4	64/4	128/4
Ceftazidime	>16	>16	>16	>16	>16	>16
Cefepime	>32	>32	>32	>32	>32	>32
Ceftriaxone	>32	>32	>32	>32	>32	>32
Aztreonam	>16	>16	>16	>16	>16	>16
Imipenem	>8	>8	>8	>8	>8	>8
Meropenem	>8	>8	>8	>8	>8	>8
Gentamicin	8	8	4	8	4	8
Tobramycin	>8	>8	>8	>8	>8	>8
Amikacin	<8	32	16	>32	16	32
Tetracycline	>8	>8	>8	>8	>8	>8
Minocycline	<1	<1	<1	4	<1	<1
Tigecycline	<1	8	2	4	<1	<1
Ciprofloxacin	1	2	2	>2	2	1
Levofloxacin	<1	8	<1	>8	<1	<1
TMP/SMX	>4/76	>4/76	>4/76	>4/76	>4/76	>4/76

*BAL, bronchoalveolar lavage; CHF, congestive heart failure; CVC, central venous catheter; TMP/SMX, trimethoprim/sulfamethoxazole; TZP, piperacillin/tazobactam. †Light gray shading indicates intermediate susceptibility; dark gray, susceptible isolates; no shading, resistant isolates.

Most *E. bruuniana* isolates were resistant to β-lactams, β-lactam and lactamase inhibitors, carbapenems, aminoglycosides, and trimethoprim/sulfamethoxazole (Table). All isolates were susceptible to minocycline, 4 (67%) to tigecycline and levofloxacin, and 2 (33%) to ciprofloxacin. The antimicrobial susceptibility patterns we found are similar to those of other *Elizabethkingia* spp. identified in previous studies (*7**–**10*). For example, reports from the United States, Hong Kong, and South Korea have revealed that *E. anophelis* and *E. meningoseptica* were frequently resistant to most β-lactams, including ceftazidime, ceftriaxone, and imipenem, but showed variable susceptibility to piperacillin/tazobactam, cefepime, ciprofloxacin, and levofloxacin (*7**–**10*).

To investigate the association between target gene mutations and fluoroquinolone resistance, we examined the mutations present in quinolone resistance–determining regions in these 6 isolates. We did not find nonsynonymous substitutions in the quinolone resistance–determining regions of *gyrA*, *gyrB*, *parC*, and *parE*, which suggests that mutations in these genes are not the cause of fluoroquinolone resistance.

In summary, our study demonstrates the clinical manifestations of *E. bruuniana* infection and the molecular characteristics of the pathogen. Because cases in our study were limited in number, further large-scale studies are necessary to investigate the antimicrobial susceptibility patterns of *E. bruuniana* and elucidate the clinical characteristics and treatment of *E. bruuniana* infection.

Appendix Additional information on *Elizabethkingia bruuniana* infections in humans, Taiwan, 2005–2017.
