# Supplementary material for: Elizabethkingia bruuniana Infections in Humans, Taiwan, 2005–2017
Source: Emerg Infect Dis. 2019 Jul;25(7):1412–4. doi: 10.3201/eid2507.180768 (PMC6590768; doi:10.3201/eid2507.180768)
Supplement: Appendix — Additional information on Elizabethkingia bruuniana infections in humans, Taiwan, 2005–2017. [file 18-0768-Techapp-s1.pdf]

Article DOI: https://doi.org/10.3201/eid2507.180768

Elizabethkingia bruuniana Infections in Humans, Taiwan, 2005–2017

Appendix

Antimicrobial Susceptibility Testing

The MICs of antimicrobial drugs were evaluated by using the broth microdilution method with Sensititer 96-well panels per the manufacturer's instructions (Thermo Fisher Scientific/Trek Diagnostics Systems, Oakwood Village, OH, USA). The susceptibilities were interpreted according to the criteria of other non-*Enterobacteriaceae* in the Clinical and Laboratory Standards Institute (CLSI) guidelines (*1*). There are no established CLSI interpretive criteria of other non-*Enterobacteriaceae* to tigecycline. Therefore, the susceptibility of tigecycline was interpreted according to the *Enterobacteriaceae* susceptibility breakpoints of the US Food and Drug Administration (susceptible MIC $\leq$2 mg/L, intermediate MIC 4 mg/L, resistant MIC $\geq$8 mg/L) (*2*).

Conditions of PCR Amplification for Quinolone-Resistance Determining Regions

The conditions for amplification of quinolone resistance–determining regions were an initial extended denaturation step of 5 min at 94°C; 30 cycles of 30 s at 94°C, 30 s at 55°C, and 1 min at 72°C; a final extension at 5 min at 72°C; and then 5 min at 4°C.

References

1. Clinical and Laboratory Standards Institute. Performance standards for antimicrobial susceptibility testing; twenty-sixth informational supplement (M100-S26). Wayne (PA): The Institute; 2016.

2. Kelesidis T, Karageorgopoulos DE, Kelesidis I, Falagas ME. Tigecycline for the treatment of multidrug-resistant *Enterobacteriaceae*: a systematic review of the evidence from microbiological and clinical studies. J Antimicrob Chemother. 2008;62:895–904. PubMed http://dx.doi.org/10.1093/jac/dkn311

3. Kumar S, Stecher G, Tamura K. MEGA7: Molecular Evolutionary Genetics Analysis version 7.0 for bigger datasets. Mol Biol Evol. 2016;33:1870–4. PubMed http://dx.doi.org/10.1093/molbev/msw054

Appendix Table. Primers for amplification and sequencing of quinolone resistance–determining regions in this study

Primer	Sequence, 5′→3′	Amplicon size, bp	Strain, GenBank accession no.
gyrA-f	GGTTATCGTGTCCAGAGCG	446	*E. bruuniana* G0146^{T}, CP014337
gyrA-r	CCGCAATACCGGAAGTACCA		
gyrB-f	ATACGCACGAAGGAGGTACG	847	
gyrB-r	CGCTCTTTCTCGTTCCATGC		
parC-f	TGGTTTCTGGATTATGCCTCTT	446	
parC-r	CCTACACCAATCCCTTCTACTCC		
parE-f	GAGCTTTCCGGTATTCAG	571	
parE-r	CACCAAGTCCCTTGAATC		

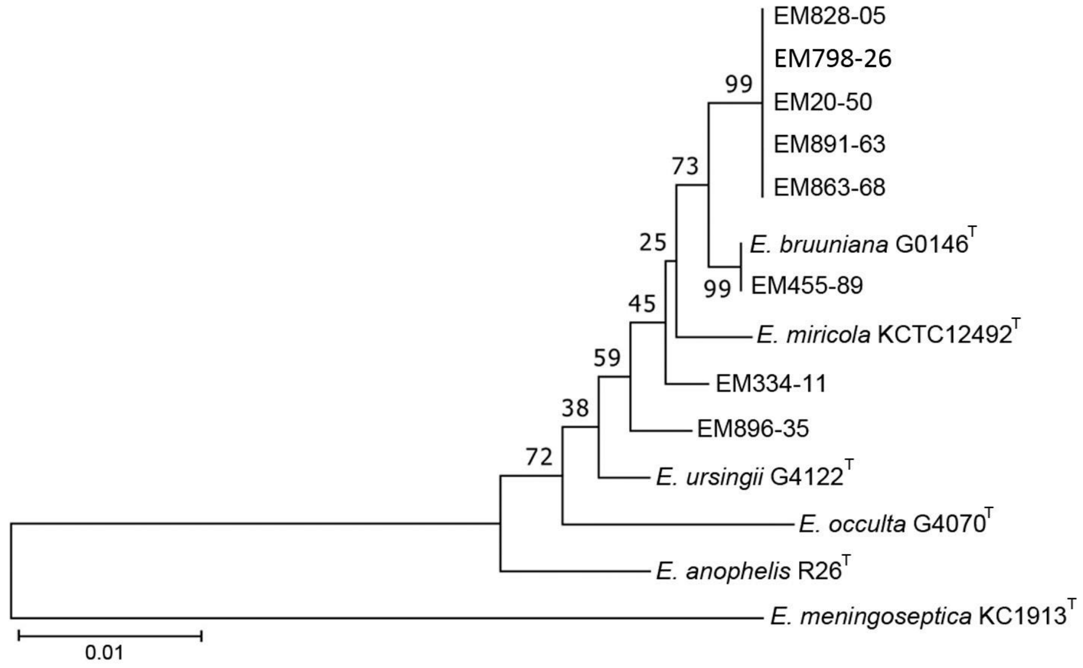

Appendix Figure 1. Phylogenetic analysis of the *rpoB* gene (positions 1,939–3,629) of *Elizabethkingia* genus type strains and isolates in this study. The phylogenetic tree was constructed by the maximum-likelihood method based on the JC69 model using MEGA 7 (*3*). The percentage of replicate trees in which the associated taxa clustered together in the bootstrap test of 1,000 replicates are shown next to the branches. The tree is drawn to scale, with branch lengths in the same units as those of the evolutionary distances used to infer the phylogenetic tree. The GenBank accession numbers of the type strains are shown as follows: *E. meningoseptica* KC1913^{T}, CP014338; *E. anophelis* R26^{T}, CP023401; *E. miricola* KCTC 12492^{T}, NZ_FLSS01000007; *E. bruuniana* G0146^{T}, CP014337; *E. ursingii* G4122^{T}, LNOK01000023; and *E. occulta* G4070^{T}, NZ_MAHX01000006.

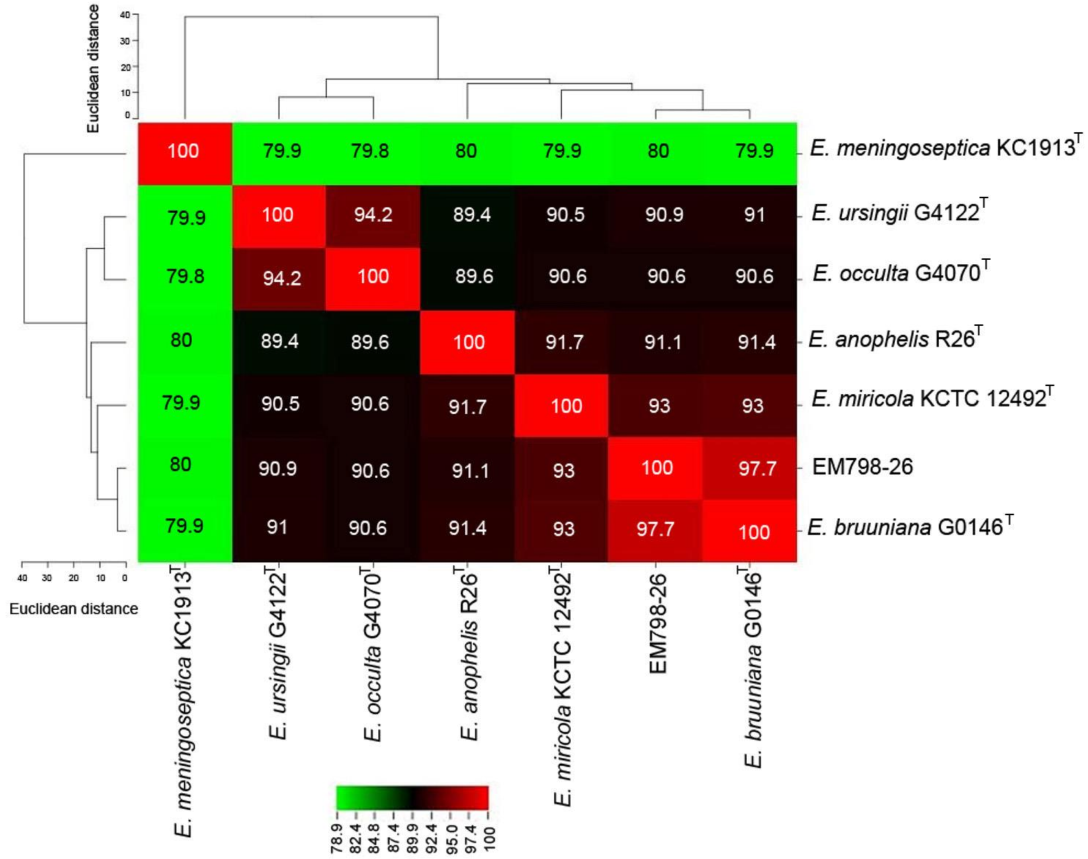

Appendix Figure 2. Average nucleotide identity between *Elizabethkingia* EM798-26 and type strains of the *Elizabethkingia* genus. The average nucleotide identity value between EM798-26 and *E. bruuniana* $G0146^T$ is 97.7%.

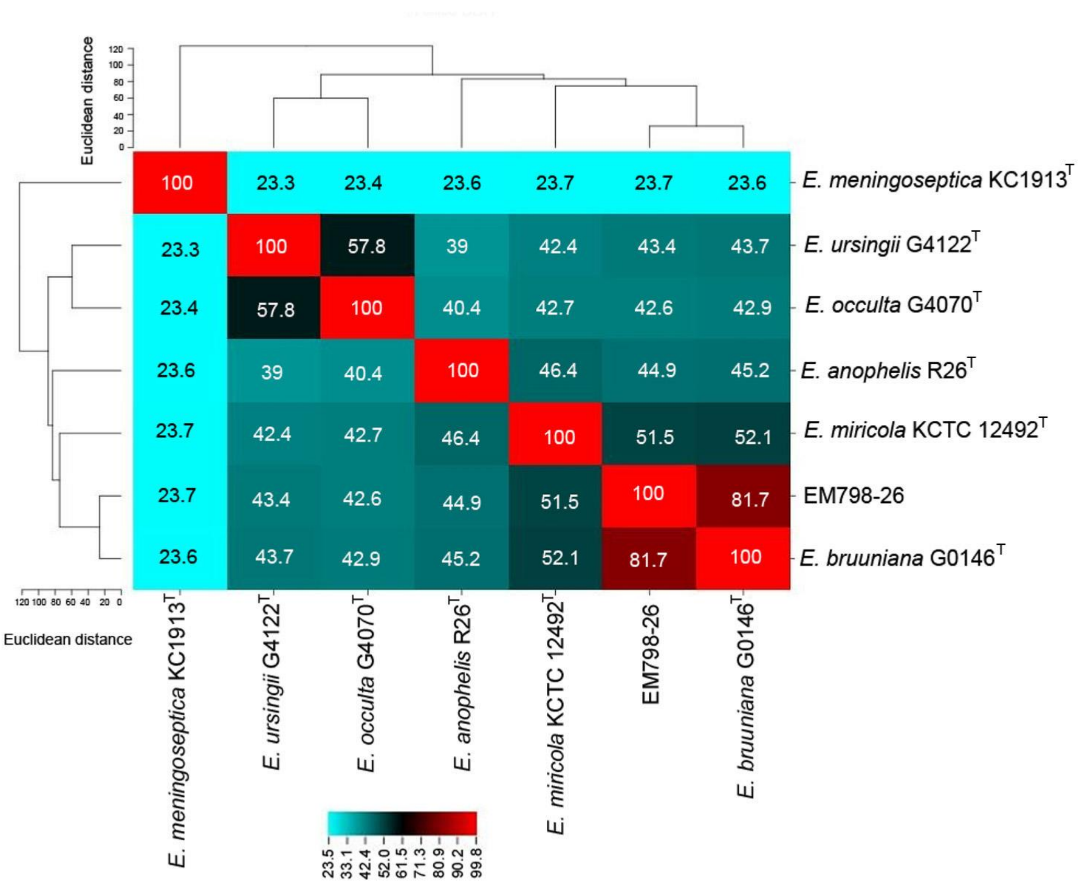

Appendix Figure 3. The in silico DNA-DNA hybridization between *Elizabethkingia* EM798–26 and type strains of the *Elizabethkingia* genus. The DNA-DNA hybridization value between EM798-26 and *E. bruuniana* G0146^T is 81.7%.
